# Early Hospital Discharge on Day Two Post-Robotic Lobectomy with Telehealth Home Monitoring

**DOI:** 10.3390/jcm13206268

**Published:** 2024-10-21

**Authors:** Giuseppe Mangiameli, Edoardo Bottoni, Alberto Tagliabue, Veronica Maria Giudici, Alessandro Crepaldi, Alberto Testori, Emanuele Voulaz, Umberto Cariboni, Emanuela Re Cecconi, Matilde Luppichini, Marco Alloisio, Debora Brascia, Emanuela Morenghi, Giuseppe Marulli

**Affiliations:** 1 Division of Thoracic Surgery, IRCCS Humanitas Research Hospital, Via Manzoni 56, Rozzano, 20089 Milan, Italy; 2 Department of Biomedical Sciences, Humanitas University, Via Rita Levi Montalcini 4, Pieve Emanuele, 20090 Milan, Italy; 3 Biostatistics Unit, IRCCS Humanitas Research Hospital, Via Manzoni 56, Rozzano, 20089 Milan, Italy

**Keywords:** enhanced recovery after surgery, telemedicine, telehealth home monitoring, thoracic surgery, robotic surgery, lung cancer, NSCLC

## Abstract

**Background:** Despite the implementation of enhanced recovery programs, the reported average postoperative length of stay after robotic lobectomy remains as 4 days. In this prospective study, we present the outcomes of early discharge (on day 2) with telehealth home monitoring device after robotic lobectomy for lung cancer in selected patients. **Methods:** All patients with a caregiver were discharged on postoperative day 2 (POD 2) with a telemonitoring device provided they met the specific discharge criteria. Inclusion criteria: <75 years old, stage I-II NSCLC, with caregiver, ECOG 0–2, scheduled for lobectomy, logistic proximity to hospital (<60 km); intra-postoperative exclusion criteria: conversion to open surgery, early complications needing hospital monitoring or redo-operation, difficult pain management, <92 HbO2% saturation on room air or need for O2 supplementation, altered vital or laboratory parameters. Teleconsultations were scheduled as follows: the first one in afternoon of POD2, two on POD3, then once a day until chest tube removal. After discharge, patients recorded their vital signs at least four times a day using the device, which allowed two surgeons to monitor them via a mobile application. In the event of sudden changes in vital signs or the occurrence of adverse events, patients had access to a direct phone line and a dedicated re-hospitalization pathway. The primary outcome was safety, assessed by the occurrence of post-discharge complications or readmissions, as well as feasibility. Secondary outcomes: comparison of safety profile with a matched control group in which the standard of care and the evaluation of resource optimization were maintained and economic evaluation. **Results:** Between July 2022 and February 2024, 48 patients were enrolled in the present study. Six patients (12.5%) dropped out due to unsatisfied discharge criteria on POD2. Exclusion causes were: significant air leaks (n:2) requiring monitoring and the use of suction device, uncontrolled pain (n:2), atrial fibrillation, and occurrence of cerebral ischemia (n:1 each). The adherence rate to vital signs monitoring by patients was 100%. A mean number of four measurements per day was performed by each patient. During telehealth home monitoring, a total of 71/2163 (1.4%) vital sign measurements violated the established acceptable threshold in 22 (52%) patients. All critical violations were managed at home. During the surveillance period (defined as the time from POD 2 to the day of chest tube removal), a persistent air leak was recorded in one patient requiring readmission to the hospital (on POD 13) and re-intervention with placement of a second thoracic drainage due to unsatisfactory lung expansion. No other postoperative complication occurred nor was there any readmission needed. Compared to the control group, the discharge gain was 2.5 days, with an economic benefit of 528 €/day (55.440 € on the total enrolled population). **Conclusions:** Our results confirm that the adoption of telehealth home monitoring is feasible and allows a safe discharge on postoperative day two after robotic surgery for stage I-II NSCLC in selected patients. A potential economic benefit (141 days of hospitalizations avoided) for the healthcare system could result from the adoption of this protocol.

## 1. Introduction

Minimally invasive surgery is widely regarded as the established therapeutic approach for patients diagnosed with early-stage non-small cell lung cancer (NSCLC), thanks to the significant benefits demonstrated in recent randomized clinical trials [1].

In the last two decades, the integration of minimally invasive surgical techniques (such as VATS and RATS lobectomy) with enhanced recovery protocols after thoracic surgery (ERAS) has aimed to decrease hospital length of stay (LOS), as well as complications and readmission rates [2,3]. Nonetheless, the currently reported postoperative length of stay following robotic lobectomy for NSCLC is approximately four days, even in highly specialized thoracic surgery centers [4,5,6], with no experience of early discharge reported in the literature. Similarly, few studies have investigated the possibility of early discharge after VATS lobectomy, with the Danish group reporting 46% of patients discharged at postoperative day (POD) 2 in their historical series [7].

We recently presented the preliminary findings of our early discharge protocol utilizing telemonitoring subsequent to robotic lobectomy for lung cancer.

Our results indicate that integrating telemedicine into an accelerated recovery protocol enables safe discharge by the second postoperative day, while also suggesting potential economic benefits for healthcare systems, if these findings are validated by studies involving larger sample sizes [8].

Herein, we report feasibility and safety results of a cohort of 48 enrolled patients compared to a matched control of patients who underwent robotic lobectomy for NSCLC in our department.

## 2. Methods

### 2.1. Study Design and Patients

In this single-center quality improvement study, all patients underwent robotic lobectomy for NSCLC at IRCCS Humanitas Research Hospital’s division of Thoracic Surgery and were discharged on POD 2 with telehealth home monitoring (ADITECH/ADiLife device, Ancona, Italy). As reported in our recently published pilot study, we have chosen POD2 as a threshold value for discharge because the greatest number of early postoperative complications such as bleeding, postoperative arrhythmia (atrial fibrillation), oxygen dependency and uncontrolled pain are able to be excluded by then [8].

Herein, we report a series of 48 patients enrolled between July 2022 and February 2024. Continuing inclusion in our protocol was possible after the publication of our preliminary data of 10 patients in which the suspension threshold of 20% readmission rate has not been reached. This study received approval from the Ethical Committee of the IRCCS Humanitas Research Hospital (research register number #201900432) and adhered to the SQUIRE guidelines as well as to the Declaration of Helsinki [9]. Informed consent was obtained from all participants in the study. Patient characteristics were collected at the face-to-face baseline assessments. Clinical and surgical data were gathered from medical records, including in-hospital and post-discharge complications within 30 days following surgery, hospital readmissions within 30 days after surgery, and the timing of both post-discharge complications and hospital readmissions.

### 2.2. Inclusion and Exclusion Criteria

All enrolled patients met the following inclusion criteria: a ECOG 0–2 performance status, age < 75 years, confirmed histopathological diagnosis of a NSCLC clinically staged as I, scheduled for lobectomy, availability of internet access, a smartphone and a caregiver living with the patient during the study, and logistic proximity to hospital (<60 km). All patients underwent robotic lobectomy according to our described technique [10].

Intra- and postoperative exclusion criteria were as following: intraoperative conversion to open surgery due to adherences or major complications (bleeding > 2000 mL, anesthesiologic complications requiring reintubation after surgery or surveillance in the intensive care unit), the occurrence of early postoperative complications needing hospital monitoring or redo-operation, difficult pain management (Numerical Rating Scale > 7), the occurrence of a postoperative complication preventing a safe discharge (i.e., persistent air leaks with incomplete lung re-expansion), a peripheral oxygen saturation <92% requiring O2 supplementation, altered vital or laboratory parameters (i.e., a systolic blood pressure <95 or >160 mmHg, body temperature >37 °C, heart rate >100 bpm).

### 2.3. Protocol

As previously reported, a specific enhanced recovery protocol covering specific items of pre-admission, admission, intraoperative care, and postoperative care was embraced [8].

The telehealth home monitoring protocol and the function of the device were explained during surgical and anesthesiologic outpatient visits systematically performed before hospital admission, thus offering pre-operative counselling to reduce fear and anxiety regarding their role in the study. All enrolled patients started respiratory physiotherapy at least one week before surgery thanks to multimedia information containing explanations for procedures downloaded by a QR code. All surgical procedures were carried out under general anesthesia with a Da Vinci Xi system (Intuitive Surgical, Sunnyvale, CA, USA). A lung-protective ventilation technique was adopted (tidal volume 4–6 mL/kg of predicted body weight, positive end-expiratory pressure–PEEP between 5 and 8 cm H_2_O, fraction of inspired oxygen between 0·5 and 0·8). An anesthesiologic multimodal protocol was adopted for all patients. This consisted in the execution of a preoperative erector spinae plane (ESP) block (levobuvicaine 0·25% 20 mL), administration of dexamethasone 4–8 mg, sulphate magnesium 1 gr before surgical incision and ketorolac 30 mg and paracetamol 1 g 30 min before awakening. In the postoperative period, pain was controlled by continuing non-steroidal anti-inflammatory drugs (ibuprofen 600 mg twice daily) and paracetamol (1000 mg three times daily). At the end of surgery, pleural drainage was ensured by placing a single 28 Fr chest tube. A Heimlich valve was systematically placed on the afternoon of POD 1. The chest tube was removed before discharge when the daily pleural effusion was less than 200 cc and no air leakage was observed.

In case of air leakage, the chest tube was left in place and the discharge was only allowed in case of adequate lung re-expansion (if the lung reaches the thoracic wall) on chest X-ray.

### 2.4. Telehealth Home Monitoring

**Figure 1:**
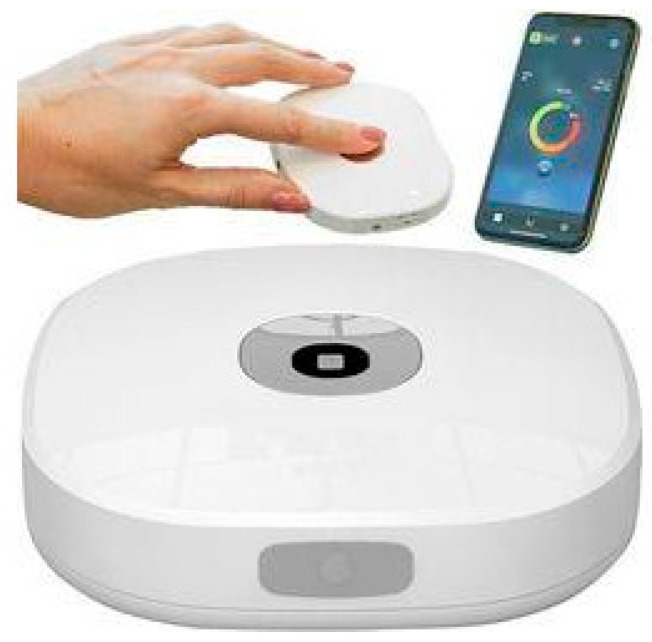
ADIBOX-HC03 Multiparametric Module.

The telehealth remote home monitoring was performed by scheduled teleconsultations and monitoring of clinical data recorded by a device (ADIBOX/ADiLife, Ancona, Italy) [11]. The ADIBOX-HC03 Multiparametric Module is a wireless Bluetooth device with class IIA medical certification that pairs with a smartphone application for data visualization (see Figure 1). The device uses infrared sensors to measure temperature and specific sensors to measure blood pressure, oxygen saturation, and heart rate.

Before inclusion in the study, all patients and their caregivers were informed on the need for the use of the device during the study period (from discharge to removal of chest drain). The device was delivered to patients the day before surgery and each patient was trained in the presence of their caregiver during hospitalization to avoid emotional stress affecting their understanding of how to correctly use the device. All clinical data recorded during the preoperative training period and the first POD was excluded from the formal analysis of this study.

Teleconsultations were scheduled as follows: first, one in the afternoon of POD2 once the patient reached his home after discharge, twice on POD3 (one in the morning and one in the afternoon), and then once a day until chest tube removal in an outpatient visit. The criterion for chest tube removal was the presence of a daily output lower than 200 cc in absence of air leakage.

After discharge, patients self-recorded their vital signs (blood pressure, body temperature, heart rate, and peripheral oxygen saturation) at least four times a day using the device. These readings were instantly accessible for review by two surgeons (research physicians) through a dedicated smartphone application. The patient was also allowed to visualize the reordered parameters.

In the event of any anomalies in the recorded vital signs (threshold violations), an alert was sent directly to the research physicians via mobile phone, including both text messages and emails.

Threshold violations were classified as moderate (yellow) and critical (red). Yellow threshold violations included the following: systolic blood pressure > 140 or <100 mmHg, diastolic blood pressure > 95 or <60 mmHg; oxygen saturation < 94%; heart rate > 100 bpm; and temperature > 37.5 °C. Red threshold violations were defined as follows: systolic blood pressure > 160 or <80 mmHg, diastolic blood pressure > 110 or <40 mmHg; oxygen saturation < 89%; heart rate > 140 bpm; and temperature > 38 °C.

In the event of red threshold violations, research physicians were alerted, prompting immediate contact with patients by telephone to obtain further information about parameter deviations and their clinical status. This procedure was also implemented in cases of missing data to provide technical assistance to the patients whenever needed.

Additionally, a direct telephone line was available to patients 24 h a day, along with a dedicated re-hospitalization pathway in the event of sudden changes in vital signs or the occurrence of adverse events. Lastly, all patients were monitored according to the standard of care following the removal of the chest drain. For all enrolled patients, a 30-day follow up was performed.

### 2.5. Outcome Measures

Primary outcomes were: (a) feasibility (rate of successful enrolment and completion of the protocol), and (b) safety (assessed through the occurrence of post-discharge complications and the number of hospital readmissions).

Secondary outcomes were: (a) comparison of results with matched controls in which the standard of care was maintained, and (b) economic evaluation (in terms of hospitalization days and costs saved).

### 2.6. Statistical Analysis

All data were collected and stored in a Microsoft Excel^®^ spreadsheet. Descriptive statistics were employed to present baseline and surgical characteristics of patients. Continuous variables are reported as mean and standard deviation, while discrete variables are reported as counts and percentages. A matched control group for safety evaluation of this protocol was obtained from a clinical cohort of patients who underwent robotic lobectomy for stage I–II NSCLC in our Department between 2019 and 2023. Each case was matched with a minimum of 1 to a maximum of 3 controls (35 cases were matched with 35 controls each, 6 cases with 2 controls each, and 1 case with 3 controls) based on the number of available patients with similar characteristics found in the database. The matching was conducted according to the following criteria: age (range of 10 years from case reference, +/− 5 years), gender, predicted post-operative FEV1 (3 groups: <70%; 70–90%; >90%), and type of lobectomy performed. Data was collected and analyzed using Microsoft Excel software (version number 16.0) and Stata version 17 (StataCorp. 2021. Stata Statistical Software: Release 17. College Station, TX, USA: StataCorp LLC), respectively. A *p*-value < 0.05 was considered statistically significant.

## 3. Results

### 3.1. Enrolment and Drop Out

From July 2022 to February 2024, 117 consecutive patients were admitted to our Division of Thoracic Surgery and underwent robotic surgery for clinical stage I-II NSCLC (no evidence of N2 diseases detected during radiological staging systematically performed by 18F-fluoride positron emission tomography). Forty-seven patients were considered to be not eligible for our protocol. The principal factors contributing to ineligibility were the following: unfit patients with an ECOG performance status greater than 2 (n = 20); age > 75 years (n = 18), living outside the 60 km range from IRCCS Humanitas Research Hospital (n = 7), and lack of a caregiver (n = 2).

Although 22 patients were potentially eligible for the study, they were not enrolled. In particular, in 18 cases the protocol had not been proposed to the patient by their tutor surgeons and four refused to participate for perceived high mental burden (n = 2) or insufficient digital skills (n = 2). Overall, 48 out of 117 patients were considered eligible and consented to participate in the study. After the patients consented to enrolment, we recorded six dropouts of the study due to the presence of intra-postoperative exclusion criteria. In particular, the causes of exclusion were: the occurrence of intraoperative bleeding more than 200 mL without need for surgical conversion to open surgery (n = 1), hemorrhagic stroke that occurred during surveillance in the recovery room (n = 1), uncontrolled surgical pain (n = 1), occurrence of a severe desaturation with need for oxygen supplementation (n = 1) on POD2, air leaks with suboptimal lung re-expansion on chest X-Ray performed on POD 2 (n = 1), and the occurrence of atrial fibrillation needing medical treatment and instrumental monitoring (n = 1) on POD2.

**Figure 2:**
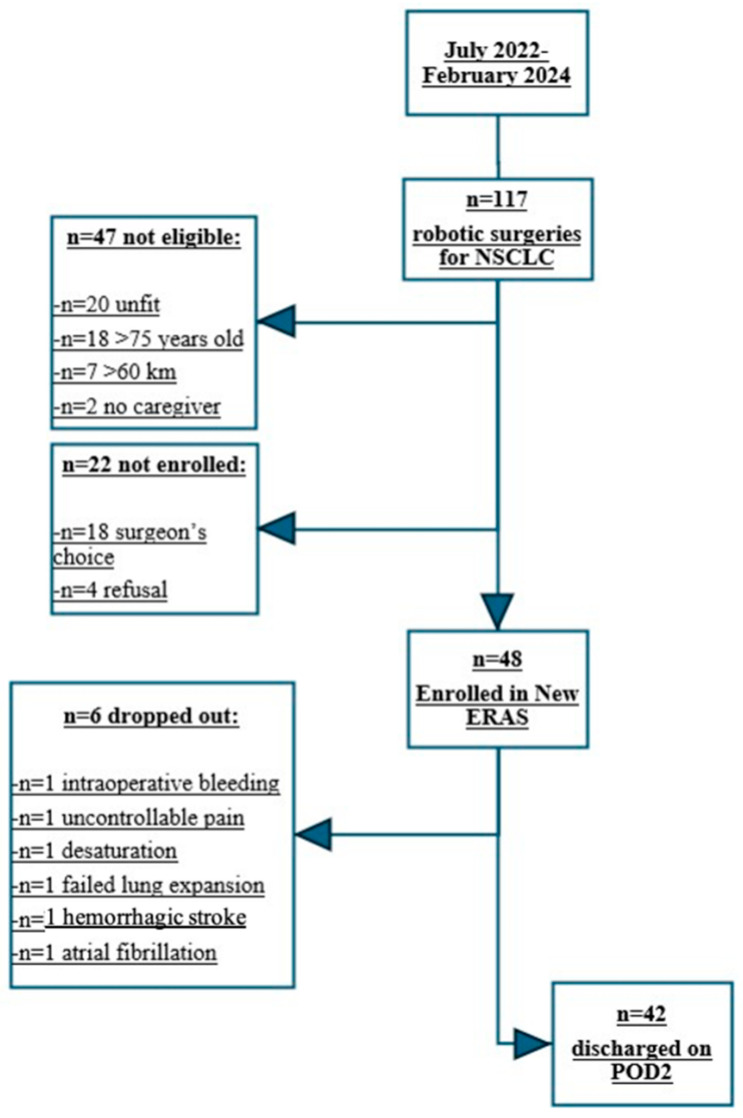
Enrolment and drop out flowchart.

In total, 42 patients participated in the study, comprising 19 males (45.2%) and 23 females (54.8%), with a mean age of 65.4 ± 7.8 years. All patients were discharged on postoperative day 2. Table 1 presents the clinical and pathological characteristics of the study population, while the flowchart depicting enrollment and dropout is summarized in Figure 2.

### 3.2. Postoperative Period

Extubation was performed for all patients at the end of surgery on the operating table.

All patients were subsequently transferred to the surgical ward after undergoing a chest X-ray in the recovery room. During the initial 36 h of hospitalization, no complications were recorded.

### 3.3. Teleconsultation and Chest Drain Management

We have not reported non-adherence to planned teleconsultations, with a total of 182 teleconsultations performed for the entire study population by two researcher surgeons. It consisted of a typical face-to-face patient-doctor interview aiming to evaluate patient status, the quality and amount of pleural effusion, and the presence/persistence and/or resolution of air leaks. Six patients were discharged on POD 2 without chest drainage while the remaining 36 patients were discharged with a chest drain connected to a Heimlich valve. In this latter group, the chest drain was removed on POD 3 in nine cases, on POD 4 in 12 cases, and after POD 4 in 15 cases due to the persistence of air leak or pleural fluid output over 200 mL/24 h (POD 5: 8, POD 6: 2, POD 7: 2, POD 8: 1, POD 12: 1, POD 15: 1).

There was no need for unplanned teleconsultations.

### 3.4. Telemonitoring of Vital Signs

**Figure 3:**
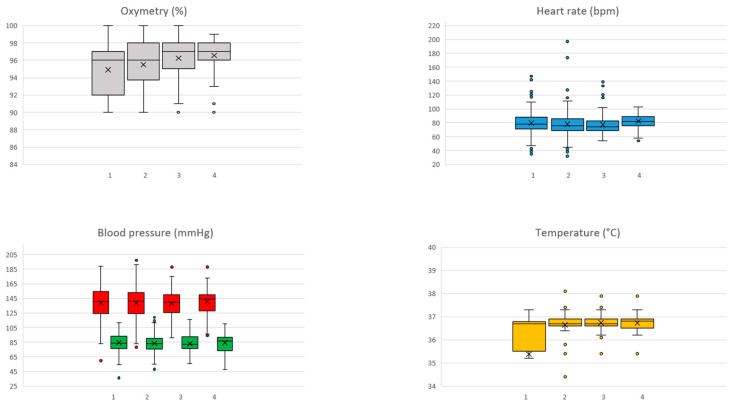
Graphical representation of measurement for each vital sign. The box is bounded by the first (bottom) and third (top) quartiles. The median is represented as a line in the box while the mean is represented as an X.

Regarding teleconsultations, the adherence rate for vital sign recording was 100%, and each enrolled patient had a mean of four measurements conducted per day. A total of 2163 sign measurements were performed with 71 (1.4%) red threshold violations recorded in 22 (52%) patients. No critical oximetry threshold violations were observed. Blood pressure threshold violation was the most common critical violation, recorded 65 times in 18 patients (43%), followed by heart rate threshold violations, recorded four times in three patients (7%). Finally, two threshold violations of temperature were recorded in two patients (5%). All threshold variations were managed by phone call. Figure 3 reports a graphical representation of measurement for each vital sign for the first four PODs; no significant variations were recorded in the following PODs.

### 3.5. Complication Rate and Readmission

During the surveillance period of early discharged patients (defined as the time from discharge on POD 2 to the day of chest tube removal), we have reported one readmission on POD 13. This patient presented with a sudden onset of subcutaneous emphysema after the removal of the chest drain the previous day. A re-hospitalization was needed to place a new thoracic drain due to unsatisfactory lung expansion on chest radiography, with patient discharge occurring after 3 days. No other postoperative complications occurred nor was any readmission needed. In the period subsequent to drainage removal, no complications or readmissions were reported within 30 days, except for one patient who presented spontaneously to the emergency department due to inadequate control of surgical site pain, which was managed conservatively with home medication.

### 3.6. Matching Analysis

The study population was ultimately compared to a control group of 50 patients who underwent robotic lobectomy for NSCLC in our department between 2019 and 2022. Data obtained are reported in Table 2. We did not detect a statistically significant difference between the two groups concerning hospital re-entry (30 days), re-intervention (30 days) and date of chest drain removal. On the other hand, an advantage concerning the number of hospitalization days saved (2.5 days) was evident with an estimated economic benefit of 528 euros per day of stay (for a total cost saving for 42 patients of 55.440 euros). To this amount, 5000 should be subtracted for the cost of the two devices which are now property of the Thoracic Surgery Division.

## 4. Discussion

In the current study, we have validated the preliminary findings from our pilot study, demonstrating that the incorporation of telehealth home monitoring within a fast-track protocol enables a safe discharge by postoperative day 2 following robotic lobectomy for early-stage NSCLC in selected patients.

As previously reported, under the input of the increased use of telemedicine emerging during the COVID-19 pandemic period, we have developed the present protocol to address two main problems for oncological surgical activity in an elective setting. The first one was the optimization of bed occupation rate in a scenario in which the resources were reduced to adapt to the need for COVID-19 patient admissions which reportedly caused a delay in oncological patient treatment worldwide. The rationale to apply a protocol of early discharge was also to reduce all nosocomial infectious risk for oncological patients during the postoperative period [12,13,14].

To the best of our understanding, this study is the first to explore the effectiveness of telemedicine in postoperative surveillance following major thoracic surgery, aiming to reduce the postoperative length of stay.

No studies are present in the literature concerning telemedicine and thoracic surgery; all the reported experiences are limited to cardiac, orthopedic, or abdominal surgery [15,16,17]. Furthermore, all studies concerning the adoption of telemedicine in surgical patients describe it exclusively as a monitoring tool during the postoperative period after a standard discharge [18,19,20,21,22,23,24,25,26,27].

Conversely, our study demonstrates that integrating telehealth home monitoring with an ERAS program significantly reduces the postoperative length of stay, decreasing from the commonly reported 4 days in centers with a high level of specialization in robotic surgery to just 2 days in our experience.

Notably, when we designed our study, we identified postoperative day 2 as the safety cutoff for discharge after major thoracic surgery. It represents the postoperative day in which we have detected the greatest number of major complications in our historical series. The occurrence of the most common medical factors preventing an early discharge (atrial fibrillation, bleeding, oxygen dependency, and uncontrolled pain) were all considered exclusion criteria. Their occurrence between the first and the second postoperative days confirmed our original hypothesis; thus, six patients were excluded from the early discharge protocol, while the remaining enrolled patients did not experience any similar complication during the postoperative period.

Only one surgical complication needed rehospitalization requiring chest drain placement. However, in this case, the complication occurred late in the postoperative period (POD 13) and it could have appeared in any patient independently of early discharge. Similarly, another patient presented to the emergency room on POD 30 due to pain which was managed with oral at home drug therapy. This data confirmed both the safety and feasibility of our protocol, considering that no other adverse events occurred during the telemonitoring period.

In the same manner, we have not identified differences in terms of number of 30-day reinterventions and 30-day re-admissions when we compared our series with matched controls in which the standard of care was maintained. Furthermore, in this latter group, the median post-operative days were similar compared to the ones commonly reported in the literature (4 days), confirming that our protocol represents a safety tool to optimize medical resources thanks to the hospitalization days saved. Furthermore, this benefit could be assessed not only as direct (through the reduction in hospitalization days) but also as indirect, due to the optimization of bed availability for additional surgical procedures.

Based our experience, the combination of robotic surgery and telemedicine with the automatic transmission of vital parameters represents a crucial ‘mixed technology’ that will be essential for enhancing and optimizing this pathway.

Our protocol (specifically tailored to robotic surgery) could allow us to decrease cost-related limitations linked to this technology and promote its widespread use [3]. Furthermore, we believe this protocol could be applied to all types of mini-invasive surgery, including VATS surgery, which is usually associated with a lower cost [28].

The systematic applicability of this approach could offer several advantages for the healthcare system as a whole.

This protocol reduces the waste of physical resources and promotes more efficient utilization of beds, meals, and medications, while also decreasing the risk of nosocomial infections by shortening the average length of hospital stay, resulting in substantial economic savings.

In the near future, there will likely be an increased adoption of telemedicine that will translate into a progressive migration of “take-care system” away from hospitals and clinics to home and mobile devices due to the widespread availability of the internet and the integration of telemedicine with in-person care [29].

However, to enhance the effectiveness and adoption of perioperative telemonitoring within the field of thoracic surgery, some present difficulties need to be overcome.

During the study period, only 3% of eligible patients refused to be enrolled due to limited availability of internet resources, insufficient digital skills, or perceived high mental burden. This amount of refusal is probably “physiological” considering that the main beneficiaries in oncological setting include those of older population and lower socioeconomic status. On the other hand, there was a 15% of failed enrolment due to surgeon’s choice, confirming a resistance and skepticism in leaving the standard of care for “innovative” technological management. Skepticism was shown by experienced surgeons in all cases and was based on their judgement and on their own clinical experience rather than objective parameters and inclusion protocol criteria.

This data highlights the need for further exploration of the current evidence regarding the effectiveness of perioperative telemonitoring interventions in thoracic surgery, as well as guidance on how to effectively implement these practices in healthcare settings. Further feasibility and usability studies as well as clinical trials will represent the prerequisites to ensure the overcome of surgical reticence and the adoption by end-users.

On the other hand, we have encountered a high availability of caregivers in supporting digital care (only in 1.7% of cases the absence of a caregiver was the reason for non-eligibility) and a high rate of adoption for both teleconsultation and the home monitoring system among the patients enrolled in the study. All planned teleconsultations were successfully completed, and patients transmitted their vital signs more frequently than required through the device, indicating that they felt central to the digital care program. This also emphasizes the importance of having a caregiver to ensure adherence throughout the process of care.

We believe that a preventive and detailed explanation of the protocol is a crucial part of a successful enrolment allowing to overcome all classic non-medical factors preventing an early discharge and the corresponding benefits.

Among these, the most frequently reported are cultural (including patient and family apprehension and insufficient organization of post-hospital care), economic (a lack of incentives for hospitals to reduce length of stay), and geographic (issues such as distance from care facilities, remote living situations, and limited availability of suitable structures for convalescence).

In this scenario, the careful selection of both patient and caregiver is pivotal to the success of this protocol. One of the major limitations of telemonitoring studies has been the tendency of a selection bias toward patients with higher education, causing reduced external validity and generalizability for widespread use [30,31]. To overcome this limitation, the solution could be to select a caregiver who lives with the patient and is capable of compensating for lack of technological abilities considering that perioperative telehealth home monitoring should not be adopted as a goal itself, but rather a support for personalized care to improve postoperative outcomes [32].

Finally, the widespread implementation of telemedicine requires an initial investment of time and resources to develop educational strategies (staring with academic educational centers), for implementation in non-academic medical centers (general practitioners and territorial medicine), and for the adoption of telehealth home monitoring both in oncological centers and within the surgical community. The results of these efforts should be translated in the optimization of general healthcare systems in a few years.

This study has some limitations. These include the sample size, a selection bias arising from the stringent selection of patients (requiring the availability of caregivers and computer literacy in elderly patients), and the fact that it represents the first study of its kind, with no prior research available on this specific topic.

## 5. Conclusions

This study corroborates our preliminary findings, demonstrating that the incorporation of telemedicine within a fast-track protocol facilitates a safe discharge on postoperative day two following robotic surgery for stage I-II NSCLC.

Careful patient selection is crucial for the success of this approach, which may be applicable across all types of minimally invasive surgery.

Additionally, our protocol highlights the potential cost saving for healthcare systems, which may be enhanced when extended to other thoracic and non-thoracic surgical interventions.

## Figures and Tables

**Table 1:** Patients’ clinical and pathological features.

Variables	Overall, n: 42
Age (years)	65.4 ± 7.8
Sex (male)	19 (45.24%)
BMI	25.6 ± 5.1
ASA score	
1	24 (57.14%)
2	6 (14.29%)
3	11 (26.19%)
4	1 (2.38%)
Smokers	
No	8 (19.05%)
Former	18 (42.86%)
Yes	16 (38.10%)
Predictive postoperative FEV1%	91.2 ± 19.1
Predictive postoperative- FVC%	92.1 ± 18.3
Tiffeneau index (FEV1/FVC) %	75.4 ± 7.0
Predictive postoperative DLCO%	82.2 ± 16.3
Predictive postoperative KCO%	89.5 ± 15.9
Histology	
Adenocarcinoma	34 (81%)
Squamous cell carcinoma	2 (5%)
Typical carcinoid tumor	6 (14%)
pTNM	
ypT0N0	2 (5%)
T1aN0	5 (12%)
T1bN0	15 (36%)
T1cN0	4 (10%)
T1cN2a	1 (2%)
T2aN0	9 (22%)
T2bN0	1 (2%)
T2bN2b	1 (2%)
T3N0	3 (7%)
T3N2a	1 (2%)
Surgery	
RUL	18 (43%)
RML	3 (7%)
RLL	5 (12%)
LUL	10 (24%)
LLL	6 (14%)
Operative time (min)	145.1 ± 38.3

RUL = Right upper lobectomy; RML: Right middle lobectomy, RLL = Right lower lobectomy; LUL: Left upper lobectomy; LLL: Left Lower lobectomy.

**Table 2:** Matching analysis.

Items	Cases (n = 42)	Control (n = 50)	*p*
Age	65.4 ± 7.8	66.4 ± 6.5	0.756
Sex (Male)	19 (45.24%)	30 (60.00%)	0.157
BMI	25.6 ± 5.1	25.5 ± 4.8	0.949
Smoke			0.404
Never	8 (19.05%)	15 (30.0%)	
Ex	18 (42.86%)	21 (42.0%)	
Active	16 (38.10%)	14 (28.0%)	0.304
FEV1% of predicted	91.2 ± 19.1	88.4 ± 19.7	0.505
FVC% of predicted	92.1 ± 18.3	96.8 ± 18.4	0.227
Tiffeneau Index (FEV1/FVC)%	75.4 ± 7.0	72.3 ± 9.9	0.088
DLCO% of predicted	82.2 ± 16.3	77.7 ± 17.9	0.212
KCO% of predicted	89.5 ± 15.9	82.9 ± 20.0	0.091
pTNM (8th edition)			0.206
IA1	5 (11.90%)	5 (10%)	
IA2	15 (37.71%)	11(22%)	
IA3	4 (9.52%)	7 (14%)	
IB	9 (21.42%)	11 (22%)	
IIA	1 (2.38%)	3 (6%)	
IIB	3 (7.14%)	10 (20)	
IIIA	3 (7.14%)	3 (6%)	
Day of discharge	2 (2–3)	4.5 (2–22)	0.001
Hospital re-entry (30 d)	2 (4.76%)	1 (2.00%)	0.590
Re-intervention (30 d)	1 (2.38%)	0	0.457
POD of drainage removal	4 (2–15)	4 (2–22)	0.112

## Data Availability

The data presented in this study are available on request from the corresponding author.
